# Impact evaluation of a community-based intervention to reduce risky sexual behaviour among female sex workers in Shanghai, China

**DOI:** 10.1186/s12889-015-1439-5

**Published:** 2015-02-14

**Authors:** Juan Liu, Liviana Calzavara, Joshua B Mendelsohn, Ann O’Leary, Laiyi Kang, Qichao Pan, Ted Myers, Jinma Ren, Yanfeng Cha, Guozheng Shi, Xiaofeng Liu, Xiuhong Tian, Huili Fan, Yinqing Ni, Robert S Remis

**Affiliations:** Dalla Lana School of Public Health, University of Toronto, Health Science Building, 155 College Street, Room 518, Toronto, ON M5T 3M7 Canada; US Centres for Disease Control, Atlanta, GA USA; Shanghai Centre for Disease Control and Prevention, Shanghai, China; Centre for Outcomes Research, University of Illinois, College of Medicine, Peoria, IL USA; Songjiang District Centre for Disease Control and Prevention, Shanghai, China; Jiading District Centre for Disease Control and Prevention, Shanghai, China; Baoshan District Centre for Disease Control and Prevention, Shanghai, China; Minhang District Centre for Disease Control and Prevention, Shanghai, China; Luwan District Centre for Disease Control and Prevention, Shanghai, China; Changning District Centre for Disease Control and Prevention, Shanghai, China

**Keywords:** China, Shanghai, Female sex worker, STI, HIV, Intervention, Condom, Risk perception

## Abstract

**Background:**

Female sex workers (FSWs) are at risk for sexually transmitted infections (STIs), including HIV. We implemented an HIV/STI preventive intervention among FSWs in Shanghai that aimed to increase condom use, improve HIV knowledge, and reduce STI and HIV incidence.

**Methods:**

From six districts in Shanghai, 750 randomly selected venue-based FSWs were allocated to either a behavioural intervention or control group. In the intervention and control groups, 221 and 278 participants, respectively, had at least one follow-up at three or six months. In analysis, we randomly selected 57 lost to follow-up cases in the intervention group and imputed baseline values to equalize the arms at n = 278 (74.1% follow-up rate in each group). The impacts of the intervention on condom use, HIV/STI risk perception and knowledge, and STI incidence were assessed using either a logistic or linear model, adjusting for the baseline measure of the outcome and venue type.

**Results:**

The intervention improved consistent condom use with any partner type in the previous month (AOR = 2.09, 95% CI, 1.43-3.04, p = 0.0001). Consistent condom use with clients in the three most recent sex acts increased in both arms, and with primary partners in the intervention arm, but there was no difference between groups after adjusting for baseline condom use and venue type. There were no differences in cumulative incidence of any STI (i.e., chlamydia, gonorrhoea, syphilis) between groups. HIV transmission knowledge (p = 0.0001), condom use skill (p = 0.0421), and self-efficacy for using condoms (p = 0.0071) were improved by the intervention. HIV-related stigma declined (p = 0.0119) and HIV and STI risk perception were improved (4.6 to 13.9%, and 9.4 to 20.0%, respectively). The intervention was associated with these improvements after adjusting for the baseline measure and venue type.

**Conclusion:**

Following a preventive intervention among Shanghai FSWs, our findings demonstrate that a simple, community-based educational intervention improved overall condom use, HIV and STI knowledge, and attitudes in relation to HIV/AIDS. The intervention should be implemented widely after tailoring educational materials regarding condom negotiation with different partner types (i.e., commercial sex clients and primary partners).

**Electronic supplementary material:**

The online version of this article (doi:10.1186/s12889-015-1439-5) contains supplementary material, which is available to authorized users.

## Background

HIV and sexually transmitted infections (STIs) have become an increasingly important public health problem in China. Reported syphilis rates increased from 0.09 per 100,000 persons in 1990 to 19.5 cases per 100,000 persons in 2008, a 200-fold increase [1]. The gonorrhoea rate increased from 7.0 per 100,000 persons in 1990 to 22.8 cases per 100,000 persons in 1999. Although this rate has since declined, it was still 1.4-fold higher in 2008 compared to 1990 [1]. Although HIV prevalence remains low (<0.1%) [2], reported new HIV diagnoses have increased dramatically each year, from 10,742 in 2007 to 39,183 in 2011. In total, 445,000 HIV diagnosed cases were cumulatively reported through 2011. As of 2011, an estimated 780,000 persons were HIV-positive [2], of whom 174,000 had AIDS. Heterosexual transmission has become the primary mode of HIV transmission in China. The proportion of newly reported HIV cases infected through heterosexual transmission has increased, from 30.6% in 2006 to 62.6% in 2011 [2]. Commercial sex appears to be a major driver of heterosexual transmission of HIV in China. A survey in 2011 found that, of 676 persons infected with HIV through heterosexual transmission, 47.6% occurred during commercial sex [3].

Although commercial sex is condemned in China, the sex work industry has taken off since the 1980s when China began its economic reform. In 2005, there were between 2.8 to 4.5 million female sex workers (FSWs) in China, and, an estimated 4-7% of men had visited an FSW in the previous year [4]. Venues in which commercial sex takes place typically include karaoke bars, night-clubs, beauty salons, hotels, massage parlours, barbershops, saunas, streets, and the back rooms of many small businesses [5]. Published studies of FSWs in China reveal that this population is vulnerable to HIV and STIs [2,3,5,6]. National sentinel surveillance has found that HIV prevalence among FSWs has remained relatively low nationally, but exceeded 1% in some settings [7]. A recent review found a high prevalence of STIs among FSWs across China: active syphilis ranged from 0.8 to 12.5% (median = 6.9%), chlamydia from 3.9 to 58.6% (median = 25.7%) and gonorrhoea from 2.0 to 85.4% (median = 16.4%) [6]. We had previously found that, among FSWs situated across six districts in Shanghai, HIV and STI prevalence were in the lower end of the typical range: HIV, 0.13%; chlamydia, 14.7%; gonorrhoea, 3.5%; and syphilis, 1.3% [8]. Consistent condom use with commercial sex clients has ranged from <10% to 55% [6, 9-15]. Low levels of condom use among FSWs have been found to be associated with low levels of perceived risk, misconceptions about HIV transmission, and lack of skills to negotiate condom use [5,9,16].

Programs for FSWs in Thailand and Africa that promoted consistent condom use and included educational components alongside testing for HIV/STIs have successfully reduced HIV and STI prevalence within these populations [17,18]. In a review of 25 HIV/STI preventive intervention studies targeting FSWs in China between 2002 to 2008 [19], with the exception of only two studies that used a quasi-experimental design, all studies had employed a pre/post design with an open cohort [14,20]. A study in Guangxi [14] used an adapted voluntary counselling and testing (VCT) program to improve consistent condom use with commercial sex clients and HIV/STI knowledge, while lowering the prevalence of any STI. An intervention in Sichuan that provided STI treatment along with health education and condom promotion was associated with increased condom use but did not reduce STI prevalence [20]. STI knowledge also increased overall but not in the low literacy group. In an early community-based intervention for FSWs in China that used a pre/post design without a control group, the use of outreach workers to deliver educational materials found increases in condom use at last sex and in the last three sexual encounters [12]. A resource-intensive, five-city community-based trial that set up new women’s health clinics and outreach teams (evaluated using a pre-post design) found that condom use at last sex improved while the prevalence of gonorrhoea and chlamydia declined [13].

The China-Canada HIV/AIDS collaborative research program responded to the absence of controlled community-based educational interventions for FSWs and other key populations in China. This multidisciplinary, multi-stakeholder program engaged government, nongovernmental organizations, and members of vulnerable populations in Shanghai. Primary aims were to study the epidemiology of HIV, other STIs, and condom use among FSWs and to rigorously evaluate a preventive intervention that aimed to increase condom use, reduce STI incidence, and improve HIV knowledge and attitudes among FSWs. The evaluation of the preventive intervention is described in the present report. Ethical approval of the study was obtained from the ethics committees of the Shanghai Municipal Centre for Disease Control and Prevention (Shanghai CDC) and the University of Toronto.

## Methods

### Study population, randomization and sample size

The 19 health districts in Shanghai were first classified as central urban core, inner suburban, or outer suburban. Two districts from each category were purposively selected and paired on the basis of most similar sociodemographic characteristics including age structure, employee annual income, proportion having completed at least a high school education, and gonorrhoea rates among women. One district from each pair was randomly allocated to receive the intervention, while the other was allocated to the control condition.

Personnel from the district health centres, especially trained for this purpose, recruited and interviewed participants for the study in May and June of 2009. A list of establishments that routinely hosted sex trade activities was prepared by each Shanghai district CDC. In the two central core districts, all establishments were included; in the suburban districts, the venue list included all venues from central and contiguous geographic areas to where venues were most heavily concentrated. We worked closely with local venue managers, local health providers, and FSWs to ensure that all candidate venues were identified and included. We classified venues with ≥15 FSWs as “larger venues”, and those with <15 FSWs as “smaller venues”. Larger venues consisted of karaoke bars and night clubs, while smaller venues included barbershops, beauty salons, foot massage parlours, massage parlours, bathhouses, and ear cleaning establishments. Risky sexual behaviours were thought to occur with greater frequency in smaller venues because FSWs in smaller venues usually earn far less than FSWs in larger venues, tend to have more commercial sex clients, and are incentivized by extra payments for unprotected sex [5,21]. We prioritized these smaller venues by recruiting 70% of the sample from smaller venues.

Within each district, we pre-identified larger and smaller venues to be included in the sampling frame. Each venue was assigned a unique identifier from a set of generated random numbers, which were then ordered in sequence to determine the order in which venues were approached. If the selected venue refused to participate, it was replaced by the following venue indicated by the ordered list. Within the larger venues, a list of FSWs was provided by the participating venue manager and a random sample of FSWs was selected. All FSWs working in the smaller venues were approached. Women were invited to participate if they reported being paid for sex, were willing to complete a questionnaire, and agreed to provide specimens for screening of HIV, syphilis, gonorrhoea and chlamydia at baseline, and at three and six month follow-up visits. Participants could use their real names or pseudonyms provided they consented to being contacted for follow-up. Consenting women were scheduled for specimen collection at a community health centre within their district or their usual work venue.

The primary outcome used to assess the impact of the intervention was condom use with commercial sex clients and primary partners. Secondary outcomes included: HIV knowledge; perceived risk; condom use self-efficacy; perceived HIV/STI stigma; and prevalence and incidence of gonorrhoea, chlamydia, syphilis and HIV. We employed conservative power estimates to identify the minimum required sample size to detect a minimum difference of 15% between the intervention and control groups on condom use with clients and primary partners. At least n = 200 in each arm was required to have adequate power (>80%; alpha = 0.05) to detect a relative risk of 1.23 and 1.43 for outcomes observed among 0.65 and 0.35 of the unexposed, respectively.

### Intervention

We developed an intervention based on strategies implemented among FSW populations in China [10,12-14], elsewhere in Asia [17,22,23], and in sub-Saharan Africa [18]. To tailor the intervention to the local setting, we conducted a multisectoral consultation during the intervention development phase with the Shanghai HIV Prevention Association, collaborators at Fudan University, venue managers, sex workers, the Shanghai Planned Parenthood Association, and public security (e.g., police). We also conducted an exploratory survey to better understand the characteristics and experiences of FSWs and to identify factors that would potentially facilitate or impede implementation of the intervention. Eleven FSWs from two districts were consulted about functional literacy, optimal duration of the educational sessions, preference about the location of the study, preferred gender of research staff, preference for group versus individual sessions, interview methods (i.e., face-to-face, paper self-administrated, or audio computer-assisted self-interview), condom use with commercial sex clients, barriers to universal condom use, and successful methods to negotiate condom use with resistant clients. We prepared a detailed manual to ensure standardization of intervention procedures.

Intervention materials included four educational video modules, each 10–15 minutes long, which covered: STI and HIV/AIDS knowledge and risk, techniques for condom use, and condom negotiation with commercial sex clients. Members of the local health district CDC with experience in HIV and STI counselling administered the questionnaire and the intervention. Research staff were trained over the course of four intensive training days. The intervention was carried out beginning one month after the baseline survey and consisted of two group counselling sessions followed by one individual counselling session, with one-week intervals between sessions. Both group sessions lasted ~90 minutes and included 6–8 participants. The content of the first group session was HIV/AIDS and STI knowledge and vulnerability enhancement, and consisted of a video, group discussion, and self-assessment of personal risk for HIV/STI infection. The second group session dealt with condom use and skills for negotiating condom use with commercial sex clients. The third individual counselling session was ~60 minutes and served to consolidate concepts from the first two sessions while providing an opportunity to practice personal condom negotiation strategies. Educational pamphlets and free condoms were distributed to both the intervention and the control groups at the time of the baseline survey.

To respond to the increase in STIs and epidemic of HIV, the Shanghai Municipal Government Office announced a systematic HIV/AIDS control and prevention action plan in 2006 that included prevention programs targeting female sex workers. Outreach workers from district CDCs routinely visited entertainment venues to distribute education materials and condoms (free or low cost), and provide free STI screens and counselling. The control districts continued to provide this routine outreach program during the study period. Free STI/HIV testing and treatment were provided to participants in both the intervention and control groups. Upon testing positive for an STI or HIV, participants were referred to a designated clinic for confirmatory testing, counselling and treatment according to Chinese national guidelines [24].

### Data sources

At baseline, three, and six months follow-up, all participants completed an interviewer-administered questionnaire, lasting ~50 minutes, which collected information on demographic characteristics, sexual history, number of commercial sex clients, condom and HIV knowledge, condom use, condom self-efficacy, and stigma. Attendance and participant satisfaction with the intervention were evaluated in the follow-up questionnaire.

During each of the three visits, urine samples were collected and tested for gonorrhoea and chlamydia using the Roche-Amplicor LCR assay (Roche, USA, http://molecular.roche.com). Blood was collected and tested for HIV and syphilis at baseline and six months. HIV antibodies were tested for using EIA (Zhu Hai Livzon Diagnostics Inc. Guangdong, China, http://www.livzon.com.cn/english). If positive, the sample was confirmed by Western blot assay (MP Biomedicals Co., USA, http://www.mpbio.com). Syphilis was detected using a rapid plasma reagin (RPR, Kehua Bio-engineering Co., Ltd., Shanghai, China, http://ft.skhb.com) and a particle agglutination test for the detection of antibodies to *Treponema pallidum* (TPPA, Fujirebio Inc, Japan, http://www.fujirebio.co.jp/english). Prevalent syphilis cases at baseline were defined by RPR titre ≥4 and reaction TPPA. Incident syphilis cases at follow-up were defined by RPR titre of a fourfold or greater increase or seroconversion after baseline and reaction TPPA [25]. STI infection was defined as any positive test for gonorrhoea, chlamydia, or syphilis.

### Measures

We asked participants if they used a condom each time they had sex in the previous month, regardless of partner type. Participants were also asked about the number of times (0–3) they had used a condom in the three most recent sex acts, with either commercial sex clients or primary partners. Consistent condom use was defined as using a condom during all three sex acts. The condom self-efficacy measure was based on three items that assessed the participant’s ability to: discuss condom use with commercial sex clients, convince a client to use a condom, and to refuse a client who did not want to use a condom in the past month [10,11]. Each item was rated from one (very incapable) to five (very capable). Cronbach’s α in our study was 0.90. A summary scale was derived, with a higher total score indicating a higher level of self-efficacy.

STI incidence was calculated among those who were negative at baseline or those who were positive at baseline and treated with antibiotics, and had at least one follow-up visit. Person-time was calculated from baseline to the second follow-up visit unless a new infection was diagnosed or there was only one follow-up visit.

HIV/STI risk perceptions were examined based on the following two questions: “What do you think is the chance that you will ever get AIDS?” and “What do you think is the chance that you will ever get an STD not including AIDS?” Response choices were “likely” or “unlikely”. Twenty-seven items from five scales were used to assess knowledge of HIV/STI and condom use: HIV transmission knowledge (10 items, Cronbach’s α = 0.76) including accurate and erroneous understanding of transmission modes; other HIV knowledge regarding treatment; vaccine and symptoms (six items, Cronbach’s α = 0.56); STI knowledge (four items, Cronbach’s α = 0.61); condom use skill (four items, Cronbach’s α = 0.48); and other condom knowledge (four items, Cronbach’s α = 0.35). The response options for knowledge items were “yes”, “no” or “do not know”. Correct answers were assigned a score of one, while incorrect answers or responses of “do not know” received a score of zero. The sum of the number of correct answers was the basis for the overall score.

Stigma toward persons with HIV was measured by using seven statements (Cronbach’s α = 0.70) related to issues around exposure to, and acceptance of, HIV-positive persons in social and professional environments and disclosure of HIV-positive status to family members and sexual partners [26]. The response options were “agree”, “disagree” or “unsure”. The stigma score was set to one if the response was “agree”, to zero if “disagree”, and 0.5 if response was “unsure”. A summary scale was formed, with a higher total score indicating a higher level of stigma.

### Data analysis

The efficacy of the intervention was assessed using logistic or linear models, controlling for the baseline measure of the outcome tested and covariates for which there was a difference between the two groups at baseline, such as venue type. Adjusted odds ratios (AOR) with 95% confidence intervals (95% CI) were obtained for binary outcomes. The adjusted mean score with 95% CI were obtained for continuous outcomes.

HIV risk reduction studies have tended to see more attrition in the control arm, a phenomenon that may be related to less staff contact with participants and weaker attachment to the study. In the present study, more women in the intervention arm were lost to follow-up compared with the control arm (41% vs 26%). To address the concern that this imbalance in follow-up, perhaps related to shame in reporting no changes in behaviour, would bias results, we took a conservative approach to primary data analysis by imputing baseline values from 57 cases randomly selected from FSWs who were lost to follow-up in the intervention group. This method served to equalize the arms at n = 278 (74% follow-up rate in each group). The present evaluation treats this imputed analysis as primary for all outcomes except STI prevalence and incidence. Non-imputed analyses are presented in Additional file 1.

We also performed a simple mediation model to assess whether intervention effects on condom use was mediated by condom self-efficacy after adjusting for baseline condom use, baseline condom self-efficacy and venue type (SAS version of ‘PROCESS’ software) [27]. The 95% bias-correct bootstrap CI (with bootstrap = 10,000) was used to determine if the intervention had an indirect effect on condom use through condom self-efficacy at follow-up (i.e., if zero was outside of the 95% bootstrap CI). Finally, we used relative risks (RR) to evaluate any differences between groups at follow-up among participants who answered knowledge questions incorrectly or reported inconsistent condom use at baseline.

All statistical analyses were carried out using SAS 9.2 for Windows (SAS Institute Inc., USA). We elected not to apply a p-value correction for multiple hypotheses (e.g., Bonferroni) as we were interested in the individual null hypotheses for each of our outcomes and were equally concerned about type I and type II error rates [28].

## Results

### Study population

**Figure 1:**
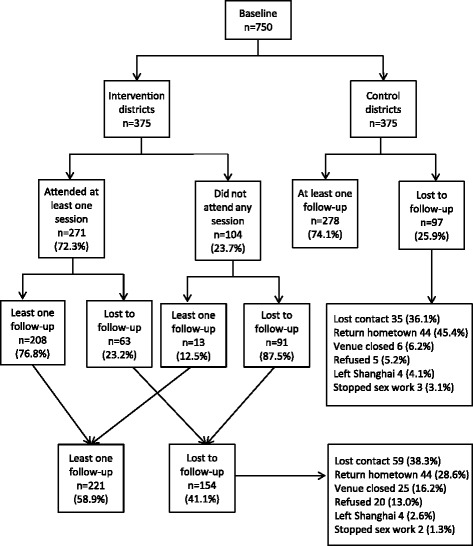
**Flow chart of intervention and follow-up among 750 FSWs in Shanghai, 2009-10.**

Of 347 pre-identified establishments within the six participating districts, 196 were approached and invited to participate, and 72% (141/196) agreed to participate. Of the 1,283 FSWs known to work at the participating venues, 848 FSWs were present at their venue during a visit by the study team and were invited to participate in the study. Of these, 88% (750/848) agreed to participate and were allocated to either the intervention or control arm based on their district location. After six months, 67% (499/750) of FSWs had at least one follow-up. Follow-up rates were 59% (221/375) in the intervention arm and 74% (278/375) in the control arm. In the intervention arm, 6% (13/221) among those with follow-up and 59% (91/154) among those who were lost to follow-up did not attend any intervention session. The main reasons participants were lost to follow-up included loss of contact with the study (38%), returned to their home town (35%), venue was closed down (12%), and refusal to attend (10%). Five women (2%) had ceased engaging in sex work altogether (Figure 1).

To assess potential contamination between arms, participants were asked about their current work venue during their follow-up interview. Among women who were present at follow-up, 87% (435/499) continued to work in the same venue as they had during the baseline survey, 2% had ceased engaging in sex work (one woman in the control arm and 11 in the intervention arm), and 10% (52/499) had changed their working venue. Of those who had changed their working venue, 92% (48/52) still worked in the same district in which they had worked at baseline. Three women in the intervention arm moved to a venue situated in a non-participating district while one woman in the control group moved to a venue situated in a different control district.

### Participation

Among 278 FSWs in the intervention group, 81% attended all three intervention sessions, 67% watched all four videos, and 17% did not attend any intervention session. Among 208 participants who attended at least one session: 99% of participants reported that the time scheduled for the session was convenient, 88-93% (across the sessions) reported that the session length was acceptable, 88-92% reported that the content of each session and video were easy to understand, and one-third reported that the content of sessions and videos were meaningful. Over half (55%) of 278 FSWs in the control group reported that they obtained HIV/AIDS information from an education pamphlet or other sources since the baseline interview.

### Baseline

Table 1 presents the comparison of the demographic and behavioural characteristics of participants in the intervention and control groups at baseline. There were no statistically significant differences between groups with respect to age, education, monthly income, ethnicity, time in Shanghai, and duration of sex work at baseline. However, more participants in the intervention group worked in barbershops and larger venues (e.g., KTV and night clubs), and more participants in the control group worked in foot massage parlours.

**Table 1:** **Baseline demographic and behavioural characteristics of 556 FSWs** ^**a**^ **in Shanghai, 2009**

		**Intervention, n (%)**	**Control, n (%)**
		**(n = 278)**	**(n = 278)**
Age (years)	Mean (IQR)	27.8 (22–33)	27.7 (21–33)
	16-19	24 (8.6)	25 (9.0)
	20-24	78 (28.1)	98 (35.3)
	25-29	80 (28.8)	43 (15.5)
	30-34	45 (16.2)	59 (21.2)
	35-39	32 (11.5)	33 (11.9)
	40+	19 (6.8)	20 (7.2)
Ethnicity	Han	265 (95.3)	266 (96.7)
	Other	13 (4.7)	9 (3.3)
Education (years)	Mean (IQR)	8.0 (6–9)	8.2 (6–9)
	None	5 (1.8)	6 (2.2)
	Elementary	65 (23.4)	71 (25.5)
	Junior high	157 (56.5)	145 (52.2)
	High school	48 (17.3)	51 (18.3)
	College or above	3 (1.1)	5 (1.8)
Marital status	Single, never married	66 (23.9)	80 (28.8)
	Cohabiting	42 (15.2)	45 (16.2)
	Married/remarried	138 (50.0)	123 (44.2)
	Separated/divorced/widowed	30 (10.9)	30 (10.8)
Annual income (USD^b^)	Mean (IQR)	7280 (4800–8640)	7600 (4800–8640)
	$0 - $3839	65 (23.6)	62 (22.9)
	$3840 - $5759	75 (27.3)	88 (32.5)
	$5760 - $7679	46 (16.7)	41 (15.1)
	$7680 - $9599	37 (13.5)	17 (6.3)
	$9600 - $19199	36 (13.1)	41 (15.1)
	$19200 or more	16 (5.8)	22 (8.1)
Length in Shanghai (months)	Mean (IQR)	34.6 (7–56)	38.0 (10–55)
Duration of sex work (months)	Mean (IQR)	20.9 (6–24)	16.6 (6–24)
Number of clients per week	Mean (IQR)	9.3 (3–14)	7.3 (3–9)
Venue type^c^	KTV/night club/spa	83 (29.9)	59 (21.2)
	Barbershop	116 (41.7)	102 (36.7)
	Foot massage	66 (23.7)	101 (36.3)
	Other^d^	13 (4.7)	16 (5.8)

^a^Randomly selected 57 participants who were lost to follow-up in the intervention arm were added to equalize the sample sizes in the two arms.

^b^1 USD = ~6.25 Chinese yuan.

^c^p =0.0056.

^d^Includes beauty salon, bath house, and other small venues.

### Condom use

Findings pertaining to condom use are presented in Table 2. The proportion of FSWs who used a condom every time while having sex with any partner type (i.e., clients or regular partners) in the past month increased from 44.6% to 65.2% (p < 0.0001) in the intervention group, but not in the control group (42.6% to 47.4%, p = 0.3022). After adjustment for baseline condom use and venue type, there was strong evidence that consistent condom use in the past month with any partner type was twice as likely in the intervention group (AOR = 2.09, 95% CI, 1.43-3.04, p = 0.0001). Consistent condom use in the three most recent sex acts with commercial sex clients increased from 70.8% to 84.9% (p = 0.0001) in the intervention group, and from 72.9% to 84.3% (p = 0.0024) in the control group. However, after adjustment for baseline condom use and venue type, there was no improvement observed when comparing groups (AOR = 0.90, 95% CI 0.52-1.55, p = 0.6977). Consistent condom use in the three most recent sex acts with primary partners (e.g., husband or boyfriend) increased from 43.9% to 54.1% (p = 0.0454) in the intervention and from 39.7% to 47.2% (p = 0.141) in the control group; however, there were no differences between groups (AOR = 1.30, 95% CI, 0.84-2.00, p = 0.2373).

**Table 2:** **Condom use among 566 FSWs** ^**a**^ **at baseline and six months after intervention, Shanghai, 2009-10**

	**Intervention (n = 278)**			**Control (n = 278)**			**Intervention vs. Control**		**AOR (95% CI)** ^**b**^	**p-value**
							**p-value**			
	**Baseline, % (n)**	**Follow-up, % (n)**	**p-value**	**Baseline, % (n)**	**Follow-up, % (n)**	**p-value**	**Baseline**	**Follow-up**		
Condom use in the most recent sex act with a client	81.2 (261)	90.7 (263)	0.002	81.4 (263)	86.3 (270)	0.1532	1.000	0.1376	1.67 (0.85-3.26)	0.1344
Consistent condom use in the three most recent sex acts with clients	70.8 (257)	84.9 (271)	0.0001	72.9 (255)	84.3 (267)	0.0023	0.6627	0.9413	0.90 (0.52-1.55)	0.6977
Condom use in the most recent sex act with a primary partner	53.2 (222)	69.3 (205)	0.0011	52.8 (214)	62.4 (218)	0.0552	1.000	0.1651	1.41 (0.90-2.22)	0.1335
Consistent condom use in the three most recent sex acts with primary partners	43.9 (212)	54.1 (205)	0.0454	39.7 (209)	47.2 (218)	0.141	0.444	0.1865	1.30 (0.84-2.00)	0.2373
Used a condom every time having sex in the past month	44.6 (271)	65.2 (273)	<0.0001	42.6 (263)	47.4 (270)	0.3022	0.6939	<0.0001	2.09 (1.43-3.04)	0.0001

^a^Randomly selected 57 participants who were lost to follow-up in the intervention arm were added to equalize the sample sizes in the two arms.

^b^Adjusted odd ratio, invention group vs. control group at follow-up after adjusting for baseline level and venue type.

In the mediation analysis, after adjusting for baseline condom use, baseline condom self-efficacy and venue type, the total, direct, and indirect effects of the intervention, mediated by condom self-efficacy at follow-up were 0.85 (95% CI, 0.47-1.23, p < 0.0001), 0.78 (95% CI, 0.38-1.18 p = 0.0001), and 0.18 (95% bootstrap CI 0.06-0.33), respectively, for consistent condom use in the previous month with either clients or primary partners; 0.06 (95% CI, −0.49-0.62, p = 0.8282), −0.71 (95% CI, −1.41--0.002, p = 0.0494) and 0.40 (95% bootstrap CI, 0.17-0.67), respectively, for consistent condom use in the three most recent sex acts with clients; and 0.38 (95% CI, −0.05-0.83, p = 0.0865), 0.24 (95% CI, −0.20-0.68, p = 0.2895), and 0.17 (95%CI, 0.06-0.34), respectively, for consistent condom use in the three most recent sex acts with primary partners.

Among those with inconsistent condom use in the three most recent sex acts with clients at baseline, the proportion using condoms consistently at follow-up was higher in the intervention when compared with the control group (69.4% vs 50.0%, RR = 0.39, 95%CI, 1.04-1.86, p = 0.0342). However, among those with inconsistent condom use in the three most recent sex acts with a primary partner at baseline, there was no difference between the two groups (38.7% vs 38.0%, RR = 1.02, 95% CI, 0.73-1.43, p = 1.000). Among those with inconsistent condom use in the previous month (either with commercial sex clients or primary partners) at baseline, there was weak evidence that the proportion using condoms consistently at follow-up was higher in the intervention group (49.0% vs 37.7%, RR = 1.30, 95% CI, 1.00-1.70, p = 0.0665).

### Condom self-efficacy

The mean score of condom self-efficacy significantly increased in both groups, but the improvement was higher in the intervention group in comparison to the control group (↑1.4 vs ↑0.6, p = 0.0219) (Table 3). After adjusting for baseline condom efficacy score and venue type, the mean condom efficacy score at follow-up in the intervention group was higher when compared with the control group (13.4 vs 12.7, p = 0.0071). Among FSWs with low reported self-efficacy at baseline, there was a statistically significant improvement among those who discussed condom use with a client (69.4% vs 44.3%), convinced a client to use a condom (68.4% vs 42.3%), and refused a client who did not want to use a condom (75.0% vs 39.4%; RR 1.57-1.90, all p < 0.001).

**Table 3:** **Mean scores of HIV/STI, condom knowledge, self-efficacy, stigma among 556 FSWs** ^**a**^ **at baseline and 6 months after intervention, Shanghai, 2009-10**

	**Intervention (n = 278)**			**Control (n = 278)**			**Intervention vs. Control**		**Adjusted mean score (95%CI) at follow-up** ^**b**^		
							**p-value**				
	**Baseline, mean score (n)**	**Follow-up, mean score (n)**	**p-value**	**Baseline, mean score (n)**	**Follow-up, mean score (n)**	**p-value**	**Baseline**	**Follow-up**	**Intervention**	**Control**	**p-value**
HIV transmission (max = 10)	6.92 (277)	8.69 (278)	<0.0001	7.44 (276)	8.23 (276)	<0.0001	0.004	0.0035	8.83 (8.57-9.08)	8.22 (7.97-8.47)	0.0001
Other HIV knowledge (max = 6)	3.47 (277)	4.56 (278)	<0.0001	3.22 (276)	3.80 (277)	<0.0001	0.0514	<0.0001	4.44 (4.23-4.65)	3.85 (3.64-4.06)	<0.0001
STI knowledge (max = 4)	3.05 (278)	3.59 (278)	<0.0001	3.10 (276)	3.42 (277)	<0.0001	0.4311	0.0396	3.57 (3.46-3.68)	3.41 (3.30-3.52)	0.0156
Condom use skill (max = 4)	2.45 (277)	3.00 (277)	<0.0001	2.44 (276)	2.80 (278)	<0.0001	0.9777	0.0532	2.86 (2.74-2.99)	2.71 (2.58-2.83)	0.0421
Other condom knowledge (max = 3)	2.25 (278)	2.61 (278)	<0.0001	2.23 (275)	2.36 (278)	0.0086	0.6777	<0.0001	2.58 (2.49-2.68)	2.37 (2.28-2.46)	0.0002
Condom self-efficacy (max = 15)	11.8 (268)	13.2 (274)	<0.0001	11.9 (267)	12.5 (278)	0.0004	0.0621	0.9977	13.4 (13.0-13.8)	12.8 (12.4-13.1)	0.0071
Stigma toward persons with AIDS (max = 7)	3.02 (278)	2.51 (278)	<0.0001	3.35 (276)	3.03 (278)	0.0018	0.0302	0.0022	2.57 (2.36-2.78)	2.89 (2.69-3.10)	0.0119

^a^Randomly selected 57 participants who were lost to follow-up in the intervention arm were added to equalize the sample sizes in the two arms.

^b^Mean score after adjusting for baseline measure of the outcome and venue type.

### Knowledge related to HIV/STI and condom use

We observed significant improvements in HIV and STI knowledge and knowledge about condom use in both groups, with greater improvement in the intervention group. There was strong evidence that the mean scores for all five knowledge scales at follow-up were higher in the intervention group after adjusting for baseline knowledge and venue type (Table 3). Among those who answered incorrectly at baseline, the proportion of correct answers about ‘using cooking oil or petroleum lubricant can cause condoms to break’ was lower in both groups after the intervention (intervention 28.0% and control 36.6%, respectively, p = 0.0758), despite the fact that this point was addressed in the video and discussed in the group session.

### HIV and STI risk perception

In the intervention group, HIV risk perception increased from 4.6% to 13.9% (p = 0.0004) and STI risk perception increased from 9.3% to 20.0% (p = 0.0011). No change was observed in the control group (HIV risk perception 5.8% to 7.8%, p = 0.4702 and STI risk perception 12.8% to 8.8%, p = 0.19). Both HIV and STI risk perception at follow-up were significantly higher in the intervention group (all p < 0.05). After adjusting for baseline level and venue type, women in the intervention group were more likely to perceive themselves as having a chance of acquiring HIV/AIDS (AOR = 1.91, 95% CI, 1.03-3.51, p = 0.0372) or an STI (AOR = 2.59, 95% CI, 1.47-4.57, p = 0.001). Among those who reported no risk for STI or HIV at baseline, the proportion who perceived themselves as likely to acquire HIV or an STI at follow-up was significantly higher in the intervention group (11.9% and 16.3%) when compared with the control (6.3% and 7.1%) (RR = 1.89, 95% CI, 1.04-3.45, p = 0.0497 and 2.29, 95% CI, 1.29-4.06, p = 0.005).

### Stigma and tolerance toward persons with HIV

The mean stigma score at follow-up decreased in both the intervention group (from 3.02 to 2.51, p < 0.0001) and control group (from 3.35 to 3.03, p = 0.0018). After adjusting for baseline stigma scale and venue type, the mean stigma score was lower in the intervention group in comparison with the control group (2.57 vs 2.89, p = 0.0119) (see Table 3). Table 4 presents the changes in individual statements of stigma towards persons with HIV. There was strong evidence that the intervention reduced negative attitudes in the three statements related to accepting persons with HIV in public settings, after adjusting for baseline level and venue type. The proportion of participants in each group who would keep it a secret if a family member was infected with HIV was similar between groups.

**Table 4:** **Stigma toward persons with HIV among 556 FSWs** ^**a**^ **at baseline and at six months after intervention, Shanghai, 2009-10**

	**Intervention (n = 278)**			**Control (n = 278)**			**Intervention vs. Control**		**AOR (95% CI)** ^**b**^	**p-value**
							**p-value**			
	**Baseline, % (n)**	**Follow-up, % (n)**	**p-value**	**Baseline, % (n)**	**Follow-up, % (n)**	**p-value**	**Baseline**	**Follow-up**		
Would keep it secret if afamily member was infected with HIV	47.1 (278)	62.9 (278)	0.0002	55.1 (276)	65.1 (278)	0.0201	0.074	0.6586	1.00 (0.69-1.46)	0.9815
Would not be willing to care for a family member who became sick with HIV in her household	4.3 (277)	2.5 (278)	0.3563	6.9 (274)	4.7 (277)	0.346	0.2541	0.2514	0.58 (0.21-1.62)	0.297
Would not allow their child to be in same classroom with another child infected with HIV	54.0 (278)	42.8 (278)	0.0109	59.6 (275)	57.8 (277)	0.7182	0.2069	0.0006	0.56 (0.9-0.81)	0.0019
Would not eat in a restaurant where cook infected with HIV	78.7 (277)	58.1 (277)	<0.0001	75.0 (276)	69.8 (278)	0.2013	0.352	0.0056	0.54 (0.37-0.78)	0.0011
Would not be willing to work next to or near a person who is infected with HIV	63.4 (276)	45.7 (276)	<0.0001	69.7 (277)	61.2 (278)	0.0432	0.141	0.0004	0.57 (0.39-0.81)	0.002
Would not tell a close family member if she learned she was infected with HIV	29.9 (278)	19.8 (278)	0.008	32.0 (275)	28.4 (278)	0.4094	0.6503	0.0226	0.66 (0.43-1.02)	0.0596
Didn’t think people diagnosed with HIV should tell their sexual partners	8.3 (278)	9.7 (278)	0.6565	17.0 (276)	6.8 (278)	0.0004	0.0029	0.2812	2.09 (1.06-4.10)	0.0325

^a^Randomly selected 57 participants who were lost to follow-up in the intervention arm were added to equalize the sample sizes in the two arms.

^b^Adjusted odd ratio, invention group vs. control group at follow-up after adjusting for baseline level and venue type.

### Prevalence and incidence of STI

Prevalence of STIs at baseline and at follow-up are presented in Table 5. Of the 65 women who tested positive for chlamydia at baseline, nine received treatment within the study. Of the 14 women who tested positive for gonorrhoea, two received treatment. Although the prevalence of gonorrhoea at baseline in the intervention group was higher than in controls (5.8% vs 0.72%, p = 0.0018), there were no differences after six months follow-up and no new HIV infections were observed at follow-up (only one HIV infection had been diagnosed at baseline). No significant changes were observed in the prevalence of STIs, including chlamydia, gonorrhoea and syphilis, in either group.

**Table 5:** **STI prevalence and 95% CI** ^**a**^ **among 556 FSWs** ^**b**^ **at baseline and six months after intervention, Shanghai, 2009-10**

	**Intervention**			**Control**			**Intervention vs. Control**		**AOR (95% CI)** ^**c**^	**p-value**
							**p-value**			
	**Baseline, % (n = 278)**	**Follow-up, % (n = 150)**	**p-value**	**Baseline, % (n = 278)**	**Follow-up, % (n = 200)**	**p-value**	**Baseline**	**Follow-up**		
Chlamydia	15.8 (9.7-19.3)	11.4 (6.8-17.6)	0.272	12.2 (8.6-16.7)	9.5 (5.8-14.4)	0.4294	0.2712	0.6875	1.16 (0.57-2.33)	0.6886
Gonorrhoea	5.8 (3.3-9.2)	2.7 (0.74-6.7)	0.2335	0.72 (0.09-2.6)	3.0 (1.1-6.4)	0.0731	0.0018	1.000	0.96 (0.30-3.09)	0.9484
Syphilis	1.1 (0.2-3.1)	0.67 (0.0-3.7)	0.9177	1.4 (0.39-3.6)	2.0 (0.55-5.0)	0.725	1.000	0.3969	0.39 (0.06-2.73)	0.3433
Any STI	19.8 (15.3-25.0)	14.1 (8.9-20.7)	0.1828	14.0 (10.2-18.7)	14.5 (9.9-20.2)	0.9898	0.0897	0.9645	0.94 (0.51-1.74)	0.8398

^a^95% exact binomial confidence interval.

^b^Randomly selected 57 participants who were lost to follow-up in the intervention arm were added to equalize the sample sizes in the two arms.

^c^Adjusted odds ratio, invention group vs control group at follow-up after adjusting for baseline level and venue type, among those had a follow-up.

The cumulative STI incidence among those who were negative at baseline, or those who were positive at baseline and treated, was no different between groups (Table 6). Compared with the control group, the STI incidence rate ratio (RR) in the intervention group at follow-up was: chlamydia, RR = 1.11, 95% CI, 0.63-1.94, p = 0.7014; gonorrhoea, RR = 1.44, 95% CI, 0.56-3.79, p = 0.399; syphilis, RR = 0.0, 95% CI, 0.0-6.92, p = 0.130; and any STI, RR = 1.09, 95% CI, 0.66-1.79, p = 0.8027.

**Table 6:** **STI incidence rate among FSWs negative at baseline or positive at baseline and known to have been treated, Shanghai, 2009-10**

	**Intervention**				**Control**				**p-value**
	**N**	**New cases**	**Follow-up time (person–years)**	**Incidence per 100 person-years (95% CI)**	**N**	**New cases**	**Follow-up time (person–years)**	**Incidence per 100 person-years (95% CI)**	
Chlamydia	190	26	106.6	24.4 (15.9-35.8)	238	30	136.3	22.0 (14.9-31.4)	0.7014
Gonorrhoea	205	11	116.9	9.4 (4.7-16.8)	266	10	153.3	6.5 (3.1-12.0)	0.399
Syphilis	150	0	95.9	0.0 (0.0-3.8)	200	3	125.5	2.4 (0.49-7.0)	0.130
Any STI	211	32	118.2	27.1 (18.5-38.2)	268	39	152.9	25.5 (18.1-34.9)	0.8027

We also undertook analysis without imputation, including a comparison of baseline differences in demographic and behavioural characteristics of participants who were, and were not, lost to follow-up. Data are reported in Additional file 1.

## Discussion

In 1998, the Chinese government announced the “Chinese national medium and long-term strategic plan on prevention and control of HIV/AIDS” [29]. Since that time, prevention programs targeting FSWs have been implemented with varying degrees of active promotion at local government levels. Typical public health intervention activities have included distribution of HIV/STI educational pamphlets, distribution of free or low-cost condoms, and STI counselling and testing [19]. As few studies had evaluated these types of interventions using a controlled, community-based study design, the present intervention study makes an important contribution to the evidence-base.

In a review of 25 HIV/STI preventive intervention studies targeting FSWs in China published between 2002–2008 and prior to the start of our trial [19], all studies except for two employed a simple pre/post design with an open cohort [14,20]. One intervention strategy used an adapted voluntary counselling and testing (VCT) program that succeeded at increasing consistent condom use with commercial sex clients and HIV/STI knowledge, while lowering the prevalence of any STI [14]. A second strategy that employed mass antibiotic treatment for STI among FSWs paired with health education and condom promotion was associated with increased condom use but had no impact on STI prevalence [20]. Our intervention, which provided free HIV and STI testing, employed interactive group and individual counselling sessions, and showed a custom video that promoted condom negotiation with different types of sex clients, improved HIV and STI knowledge and condom use with all partners in the past month. FSWs allocated to the intervention group were twice as likely as those in the control group to use condoms consistently in the past month with any type of partner, including commercial sex clients and primary partners. We did not, however, find improvements in condom use when stratifying by partner type (i.e., primary partners vs commercial sex clients) and improvements in condom use were not evident in the three most recent sex acts with either commercial sex clients or primary partners. Although consistent condom use with clients increased in both the intervention and control groups at six months follow-up, there was no evidence for differences between groups. However, a mediation analysis showed that the intervention had an indirect effect on consistent condom use through increases in condom use self-efficacy at follow-up. This provided evidence that the intervention was having the desired effect among both commercial sex clients and primary partners.

Improvements associated with this intervention were consistent with other published studies [10-14,17,22,23,30-32]. Two intervention studies among FSWs have been published since our trial [33,34]. In an evaluation of an uncontrolled intervention that aimed to increase availability of condoms and voluntary counselling and testing, Shi and colleagues found that consistent condom use with commercial sex clients increased from 39.6% to 59.6% between 2006 and 2008 [33]. In a controlled intervention study for FSWs that used multimedia group counselling sessions based on information-motivation behavioural skills training, more consistent use of condoms was reported with stable partners after three months follow-up. Among non-stable partners, however, participants in the control group reported greater increases in protected sex when compared with the intervention group [34]. HIV prevention intentions with stable partners, negative attitudes towards condoms, and condom self-efficacy with non-stable partners also improved.

In relation to knowledge, the present intervention improved HIV, STI, and condom knowledge among those who answered incorrectly at baseline. Among those who answered incorrectly at baseline, the proportion that endorsed the statement: “using cooking oil or petroleum lubricant can cause condoms to break” was still low in both the control and intervention groups (28.0% and 36.6%, respectively), even though this point had been addressed in the intervention. Future interventions should emphasize and demonstrate how oil-based lubricants can cause condoms to break.

In contrast to other studies [10,12,14,18,22,23,31], we did not find a reduction in the incidence or prevalence of STIs at follow-up. Hong and colleagues found mixed results when comparing nine studies reporting STI rates with some reporting significant reductions in STI infection rates and others reporting no significant changes or even increased STIs despite more condom use [19]. A study that evaluated an outreach program targeting FSWs in Shanghai found decreased rates of bacterial vaginosis and syphilis and increases in genital warts and chlamydia over a 1.5-year period [35]. This absence of an effect on incidence and prevalence of STIs could have been related to lag times between behavior change and impact on biomarkers that may have extended past the study end date. In our study, it was not surprising that no new HIV infections were observed at follow-up given only one infection was recorded at baseline. Although HIV and STI risk perceptions were also low, more FSW in the intervention group considered themselves to be at risk compared with the control group at follow-up.

STI treatment has been a component of STI control interventions in several programs that aimed to reduce STI prevalence [36]. In a study of FSWs in China [20], there was no difference in chlamydia or gonorrhoea rates after three months of mass STI treatment and 60% of FSWs were unwilling to continue treatment. However, mass treatment appeared to prevent increases in chlamydia infections in the intervention group, when compared with controls. Our study provided treatment free-of-charge to participants who tested positive for an STI, but the uptake of treatment following baseline testing was low. Hence, the importance of treatment should be emphasized and included in educational and health promotion programs.

Stigma toward persons with HIV infection is common in China [37,38]. At baseline, stigmatizing attitudes were particularly high among participants in relation to contact with HIV-infected persons where no risk of transmission was present (i.e., in schools, within the workplace, or at restaurants). Our success in reducing these stigmatizing attitudes after six months suggests that educational programs that modify misconceptions about HIV transmission can reduce HIV-related stigma at the individual level. However, the proportion of participants who indicated that they would keep it a secret if a family member was HIV-positive was significantly higher in both intervention and control groups at follow-up. This contrasting finding is consistent with the persisting discrimination against people living with HIV in China [39].

There were several limitations to our study. FSWs are a highly heterogeneous group [5]. We targeted establishment-based FSWs including those who worked in larger venues and smaller, lower-income venues. This approach has since been validated in studies that have shown variation in HIV risk profiles by venue type [40]. Notably, “low-tier” or street-based FSWs have been found to have a greater risk of HIV infection when compared with other types of venues [40,41]. Given the focus of our intervention, and difficulties with follow-up, our study did not attempt to recruit these highly vulnerable street-based FSWs and is therefore not generalizable to this population. There is an absence of interventions among street-based FSWs in China, which need to be tailored for this unique risk environment.

Our findings may have been biased towards the null hypothesis if women who were more predisposed to changing their behaviour were also more likely to have agreed to participate in intervention activities, while women electing not to participate were less amenable to intervention activities. In the intervention arm, 83% of FSWs who participated in the baseline survey attended at least one intervention session, while 17% of participants did not attend any intervention session. Among FSWs who did not attend any intervention session in the intervention districts, 39% lost contact with the study and 31% returned to their hometown or village. Although contamination between intervention and control arms was possible, it was unlikely to have biased our findings. Only FSWs who participated in the baseline survey in the three intervention districts were scheduled to the intervention sessions within their own district. Therefore, it was very unlikely that participants from the control arm could have been accidentally admitted to intervention sessions.

Follow-up rates in our study prior to imputed analysis were 59% in the intervention group and 74% in the control group. Though less than ideal, high attrition rates have been observed in other HIV and STI-related intervention trials in China [10,14,20]. Given the potential for bias from differential attrition, we took a conservative approach to analysis by imputing outcomes from a random sample of those lost to follow-up in the intervention arm. This served to balance the follow-up rates between arms. In the non-imputed analysis (i.e., the analysis among those had a follow-up, n = 221 intervention group and n = 278 control group), we obtained similar results as in the imputed analysis, but with more improvements observed on various measures of condom use, HIV knowledge, and condom use self-efficacy.

Social and contextual factors might explain some of this differential attrition. Given that commercial sex is condemned in China, female sex workers are often subject to administrative and criminal sanctions [42]. Fear of police crackdowns and arrest may have led to frequent migration of FSWs, interfering with study follow-up. During the study, we also encountered several unexpected events that may have compounded problems with follow-up. In late May 2009, security was strengthened by the Public Security of the Government due to celebration of the 60th Anniversary of the Founding of the People’s Republic of China on October 1, 2009. Before and during this day, many venues suspected of providing commercial sex services were shut down or ceased routine commercial sex activities for fear of arrest. A total of eight venues were closed within three months after the baseline interview, including seven in intervention districts (affecting 25 participants), while only one venue was closed across the control districts (affecting 6 participants). The main reasons for loss to follow-up overall were: 38% lost contact with the study, 35% returned to their hometown, 12% saw their venue close down, and 10% declined to continue their participation.

Social desirability bias might have affected participants’ self-reported sexual behaviours and reported perception of HIV and STI risk [43]. A study among FSWs in China found that FSWs with high levels of social desirability were less likely to self-report an STI [44]. Although self-reported sexual behaviour is prone to bias and has been an inconsistent correlate of STIs, these measures are often the only means with which to evaluate the efficacy of behavioural interventions. In an early study by Zenilman and colleagues [45], condom use was not associated with STI incidence. In a secondary analysis of the Project RESPECT intervention study, which sought to identify correlates of incident STIs among STI clinic patients, there were few behavioural correlates and some were in an unexpected direction [46]. However, in a re-analysis of these data that included participants only if their visit to the STI clinic resulted from a known exposure after having been named as a sexual contact of someone with an STI, condom use was indeed associated with STI infection [47].

In relation to sustainability, we evaluated the impact of the preventive intervention over a relatively short period of six months. It is therefore unclear whether the improvements observed at follow-up could be maintained over the long term. In several studies conducted in China and elsewhere, long-term effects of behavioural interventions on condom use for FSWs have demonstrated sustained increases in consistent condom use and reductions in STI prevalence [15,17,23]. Reinforcement of intervention programs may be important for sustaining impact, and if the intervention is expanded, evaluations should be undertaken at routine intervals to ensure that any improvements are sustainable.

## Conclusions

This study assessed the effect of an intervention for FSWs, helping to inform HIV and STI prevention efforts by demonstrating that a simple, community-based educational intervention improved overall condom use, HIV and STI knowledge, and attitudes in relation to HIV and AIDS. The high level of acceptance of this intervention among venue owners bodes well for routine implementation of this intervention by local health districts. Successful scale-up in other jurisdictions in China would depend on the availability of collaborators among government agencies, especially local police departments. As commercial sex is condemned in China and sex workers may fear criminal prosecution, it can be difficult to recruit FSWs to interventions. Protecting participants must be a priority for any study. We consulted extensively with public security and other relevant government agencies in an effort to gain their support. No participants were arrested during the study period. In scaling-up this intervention, we recommend longer follow-up times and educational messages about condom use that are differentiated and tailored for commercial sex clients and primary partners. Given that scaling-up interventions draws resources away from other preventive activities, we also recommend routine evaluations to ensure that benefits are sustained over time and mark an improvement in relation to the standard of care.

## Additional file

Additional file 1: Table S1. Baseline demographic and behavioural characteristics among 499 FSWs with follow-up and those lost to follow-up, Shanghai, 2009. **Table S2.** Condom use among 499 FSWs at baseline and six months after intervention, Shanghai, 2009-10. **Table S3.** Mean scores of HIV/STI, condom knowledge, self-efficacy, stigma among 499 FSWs at baseline and six months after intervention, Shanghai, 2009-10. **Table S4.** Stigma toward persons with HIV among 499 FSWs at baseline and at six months after intervention, Shanghai, 2009-10. **Table S5.** STI prevalence and 95% CI among 499 FSWs at baseline and six months after intervention, Shanghai, 2009-10.

## Abbreviations

AOR: Adjusted odd ratio
CDC: Center for Disease Control and Prevention
CI: Confidence interval
FSW: Female sex worker
IQR: Interquartile range
RR: Relative risk
STI: Sexually transmitted infection
