# Supplementary material for: Impact evaluation of a community-based intervention to reduce risky sexual behaviour among female sex workers in Shanghai, China
Source: BMC Public Health. 2015 Feb 14;15:147. doi: 10.1186/s12889-015-1439-5 (PMC4377073; doi:10.1186/s12889-015-1439-5)
Supplement: Additional file 1: Table S1. — Baseline demographic and behavioural characteristics among 499 FSWs with follow-up and those lost to follow-up, Shanghai, 2009. Table S2. Condom use among 499 FSWs at baseline and six months after intervention, Shanghai, 2009-10. Table S3. Mean scores of HIV/STI, condom knowledge, self-efficacy, stigma among 499 FSWs at baseline and six months after intervention, Shanghai, 2009-10. Table S4. Stigma toward persons with HIV among 499 FSWs at baseline and at six months after intervention, Shanghai, 2009-10. Table S5. STI prevalence and 95% CI among 499 FSWs at baseline and six months after intervention, Shanghai, 2009-10. [file 12889_2015_1439_MOESM1_ESM.pdf]

Additional File 1

Table S1 Baseline demographic and behavioural characteristics among 499 FSWs with follow-up and those lost to follow-up, Shanghai, 2009

		Intervention		Control	
		Followed, n (%) (n=221)	Lost, n (%) (n=154)	Followed, n (%) (n=278)	Lost, n (%) (n=97)
Age (years)	Mean (IQR)	27.8 (22-33)	26.4 (22-31)	27.7 (21-33)	26.3 (22-30)
	16-19	19 (8.6)	19 (12.3)	25 (9.0)	13 (13.4)
	20-24	59 (26.7)	53 (34.4)	98 (35.3)	35 (36.1)
	25-29	64 (29.0)	38 (24.7)	43 (15.5)	24 (24.7)
	30-34	40 (18.1)	22 (14.3)	59 (21.2)	12 (12.4)
	35-39	23 (10.4)	18 (11.7)	33 (11.9)	8 (8.2)
	40+	16 (7.2)	4 (2.6)	20 (7.2)	5 (5.2)
Ethnicity (%)	Han	209 (94.6)	147 (96.1)	266 (96.7)	91 (93.8)
	Other	12 (5.4)	6 (3.9%)	9 (3.3)	6 (6.2)
Education (years)	Mean (IQR)	7.9 (6-9)	8.2 (7.5-9)	8.2 (6-9)	8.3 (7.5-9)
	None	5 (2.3)	1 (0.7)	6 (2.2)	4 (4.1)
	Elementary	54 (24.4)	33 (21.6)	71 (25.5)	19 (19.6)
	Junior high	120 (55.4)	89 (58.2)	145 (52.2)	53 (54.6)
	High school	40 (18.1)	26 (17.0)	51 (18.3)	13 (13.4)
	College or above	2 (0.9)	4 (2.6)	5 (1.8)	8 (8.2)
Marital status (%)	Single, never married	58 (26.4)	40 (26.3)	80 (28.8)	37 (38.1)
	Cohabiting	29 (13.2)	29 (19.1)	45 (16.2)	22 (22.7)
	Married/remarried	110 (50.0)	72 (47.4)	123 (44.2)	31 (32.0)
	Separated/divorced/widowed	23 (10.5)	11 (7.2)	30 (10.8)	7 (7.2)
Annual income (USD[a])	Mean (IQR)	6740 (4800-8640)	9560 (4800-7500)[b]	7600 (4800-8640)	8640 (4800-7500)
	$0 - $3839	54 (24.8)	28 (18.5)	62 (22.9)	16 (16.5)
	$3840 - $5759	64 (29.4)	30 (19.9)	88 (32.5)	35 (36.1)
	$5760 - $7679	38 (17.4)	26 (17.2)	41 (15.1)	12 (12.4)
	$7680 - $9599	29 (13.3)	10 (6.6)	17 (6.3)	6 (6.2)
	$9600 - $19199	24 (11.0)	38 (25.2)	41 (15.1)	16 (16.5)
	$19200 or more	9 (4.1)	19 (12.6)	22 (8.1)	12 (12.4)
Length in Shanghai (months)	Mean (IQR)	36.3 (7-60)	30.5 (4-47)	38.0 (10-55)	31.1 (4-48)
Duration of sex work (months)	Mean (IQR)	21.6 (6-24)	17.0 (3-24)[b]	16.6 (6-24)	15.1 (5-18)
Number of clients per week	Mean (IQR)	9.0 (3-14)	9.7 (3-15)	7.3 (3-9)	6.9 (2-8)
Venue type	KTV/night club/spa	68 (30.8)	48 (31.2)[b]	59 (21.2)	41 (42.3)[b]
	Barbershop	91 (41.2)	75 (48.7)	102 (36.7)	21 (21.6)
	Foot massage	49 (22.2)	31 (20.1)	101 (36.3)	34 (35.1)
	Other[c]	13 (5.9)	0 (0.0)	16 (5.8)	1 (1.0)

[a] 1 USD = ~6.25 Chinese yuan
[b] $p < 0.05$ comparison within groups
[c] Includes beauty salon, bath house, and other small venues

Table S2 Condom use among 499 FSWs[a] at baseline and six months after intervention, Shanghai, 2009-10

	Intervention (n=221)			Control (n=278)			Intervention vs. Control p-value		AOR (95% CI)[b]	p-value
	Baseline, % (n)	Follow-up, % (n)	p-value	Baseline, % (n)	Follow-up, % (n)	p-value	Baseline	Follow-up		
Condom use in the most recent sex act with a client	79.2 (207)	91.2 (216)	0.0008	81.4 (263)	86.3 (270)	0.1531	0.6427	0.1234	1.85 (0.91-3.75)	0.0898
Consistent condom use in the three most recent sex acts with clients	69.5 (203)	87.1 (217)	<0.0001	72.9 (255)	84.3 (267)	0.0023	0.4744	0.4536	1.10 (0.61-1.96)	0.7567
Condom use in the most recent sex act with a primary partner	52.6 (175)	73.4 (158)	0.0001	52.8 (214)	62.4 (218)	0.0552	1.000	0.0328	1.75 (1.07-2.86)	0.0249
Consistent condom use in the three most recent sex acts with primary partners	45.5 (167)	58.8 (160)	0.0223	39.7 (209)	47.2 (218)	0.1409	0.3052	0.0351	1.59 (1.00-2.51)	0.0482
Used a condom every time having sex in the past month	46.0 (215)	71.9 (217)	<0.0001	42.6 (263)	47.4 (270)	0.3023	0.557	<0.0001	2.82 (1.89-4.23)	<0.0001

[a] Includes participants who had follow-up visit: 221 participants were in the intervention group and 278 in the control group.
[b] Adjusted odd ratio, invention group vs. control group at follow-up after adjusting for baseline level and venue type

Table S3 Mean scores of HIV/STI, condom knowledge, self-efficacy, stigma among 499 FSWs[a] at baseline and 6 months after intervention, Shanghai, 2009-10

	Intervention (n=221)			Control (n=278)			Intervention vs. Control p-value		Adjusted mean score (95%CI) at follow-up[b]		
	Baseline, mean score (n)	Follow-up, mean score (n)	p-value	Baseline, mean score (n)	Follow-up, mean score (n)	p-value	Baseline	Follow-up	Intervention	Control	p-value
HIV transmission (max=10)	6.91 (220)	9.14 (221)	<0.0001	7.44 (276)	8.23 (276)	<0.0001	0.0046	<0.0001	9.20 (8.94-9.45)	8.20 (7.96-8.44)	<0.0001
Other HIV knowledge (max=6)	3.48 (220)	4.85 (221)	<0.0001	3.22 (276)	3.80 (277)	<0.0001	0.062	<0.0001	4.69 (4.47-4.92)	3.81 (3.61-4.02)	<0.0001
STI knowledge (max=4)	3.03 (221)	3.71 (221)	<0.0001	3.10 (276)	3.42 (277)	<0.0001	0.4415	<0.0001	3.68 (3.57-3.80)	3.39 (3.29-3.50)	<0.0001
Condom use skill (max=4)	2.44 (221)	3.11 (221)	<0.0001	2.44 (276)	2.80 (278)	<0.0001	0.9811	0.0011	2.97 (2.84-3.11)	2.69 (2.56-2.81)	0.0005
Other condom knowledge (max=3)	2.26 (221)	2.70 (221)	<0.0001	2.23 (275)	2.36 (278)	0.0086	0.587	<0.0001	2.67 (2.57-2.76)	2.37 (2.28-2.46)	<0.0001
Condom self-efficacy (max=15)	11.7 (211)	13.5 (217)	<0.0001	11.9 (267)	12.5 (278)	0.0004	0.0415	0.3304	13.7 (13.3-14.1)	12.7 (12.4-13.1)	0.0003
Stigma toward persons with AIDS (max=7)	3.00 (221)	2.36 (221)	<0.0001	3.35 (276)	3.03 (278)	0.0018	0.0253	<0.0001	2.43 (2.20-2.66)	2.91 (2.70-3.12)	0.0007

[a] Includes participants who had follow-up visit: 221 participants were in the intervention group and 278 in the control group.
[b] Mean score after adjusting for baseline measure of the outcome and venue type

Table S4 Stigma toward persons with HIV among 499 FSWs[a] at baseline and at six months after intervention, Shanghai, 2009-10

	**Intervention (n=221)**			**Control (n=278)**			**Intervention vs. Control p-value**		**AOR (95% CI)[b]**	**p-value**
	**Baseline, % (n)**	**Follow-up, % (n)**	**p-value**	**Baseline, % (n)**	**Follow-up, % (n)**	**p-value**	**Baseline**	**Follow-up**		
Would keep it secret if a family member was infected with HIV	46.6 (221)	66.5 (22)	<0.0001	55.1 (276)	65.1 (278)	0.020	0.0741	0.8148	1.23 (0.82-1.83)	0.3118
Would not be willing to care for a family member who became sick with HIV in her household	4.5 (220)	2.3 (221)	0.2892	6.9 (274)	4.7 (277)	0.346	0.3524	0.2293	0.53 (0.17-1.61)	0.2633
Would not allow their child to be in same classroom with another child infected with HIV	53.4 (221)	39.4 (221)	0.0042	59.6 (275)	57.8 (277)	0.7182	0.1922	<0.0001	0.49 (0.34-0.72)	0.0003
Would not eat in a restaurant where cook infected with HIV	78.3 (221)	52.5 (221)	<0.0001	75.0 (276)	69.8 (278)	0.2013	0.4531	0.0001	0.44 (0.30-0.65)	<0.0001
Would not be willing to work next to or near a person who is infected with HIV	62.9 (221)	40.7 (221)	<0.0001	69.7 (277)	61.2 (278)	0.0432	0.1339	<0.0001	0.47 (0.32-0.68)	<0.0001
Would not tell a close family member if she learned she was infected with HIV	29.4 (221)	16.7 (221)	0.0023	32.0 (275)	28.4 (278)	0.4094	0.6013	0.0031	0.55 (0.34-0.88)	0.012
Didn't think people diagnosed with HIV should tell their sexual partners	8.1 (221)	10.0 (221)	0.6189	17.0 (276)	6.8 (278)	0.0004	0.0053	0.2728	1.91 (0.96-3.81)	0.0644

[a] Includes participants who had follow-up visit: 221 participants were in the intervention group and 278 in the control group.
[b] Adjusted odd ratio, invention group vs. control group at follow-up after adjusting for baseline level and venue type

Table S5 STI prevalence and 95% CI[a] among 499 FSWs[b] at baseline and six months after intervention, Shanghai, 2009-10

	Intervention			Control			Intervention vs. Control p-value		AOR (95% CI)[c]	p-value
	Baseline, % (n=221)	Follow-up, % (n=150)	p-value	Baseline, % (n=278)	Follow-up, % (n=200)	p-value	Baseline	Follow-up		
Chlamydia	14.0 (9.7-19.3)	11.4 (6.8-17.6)	0.5638	12.2 (8.6-16.7)	9.5 (5.8-14.4)	0.4294	0.6466	0.6875	1.16 (0.57-2.33)	0.6886
Gonorrhea	5.4 (2.8-9.3)	2.7 (0.74-6.7)	0.3112	0.72 (0.09-2.6)	3.0 (1.1-6.4)	0.0731	0.0038	1.000	0.96 (0.30-3.09)	0.9484
Syphilis	0.45 (0.0-2.5)	0.67 (0.0-3.7)	0.6657	1.4 (0.39-3.6)	2.0 (0.55-5.0)	0.7256	0.3888	0.3969	0.39 (0.06-2.73)	0.3433
Any STI	17.7 (12.9-23.3)	14.1 (8.9-20.7)	0.4439	14.0 (10.2-18.7)	14.5 (9.9-20.2)	0.9898	0.3264	0.9645	0.94 (0.51-1.74)	0.8398

[a] 95% exact binomial confidence interval
[b] Includes participants who had at least one follow-up visit: 221 participants were in the intervention group and 278 in the control group.
[c] Adjusted odds ratio, invention group vs. control group at follow-up after adjusting for baseline level and venue type
